# Fpocket: An open source platform for ligand pocket detection

**DOI:** 10.1186/1471-2105-10-168

**Published:** 2009-06-02

**Authors:** Vincent Le Guilloux, Peter Schmidtke, Pierre Tuffery

**Affiliations:** 1 ICOA – Institut de chimie organique et analytique – UMR CNRS 6005, Div. of chemoinformatics and molecular modeling, University of Orléans, Orléans, France; 2 Dpto Fisicoquimica, Fac Farmacia, Univ Barcelona, Barcelona, Spain; 3 Molécules Therapeutiques in silico, INSERM, UMR-S 973, University Paris Diderot – Paris 7, Paris, France; 4 Ressource Parisienne en Bioinformatique Structurale, University Paris-Diderot, Paris, France

## Abstract

**Background:**

Virtual screening methods start to be well established as effective approaches to identify hits, candidates and leads for drug discovery research. Among those, structure based virtual screening (SBVS) approaches aim at docking collections of small compounds in the target structure to identify potent compounds. For SBVS, the identification of candidate pockets in protein structures is a key feature, and the recent years have seen increasing interest in developing methods for pocket and cavity detection on protein surfaces.

**Results:**

Fpocket is an open source pocket detection package based on Voronoi tessellation and alpha spheres built on top of the publicly available package Qhull. The modular source code is organised around a central library of functions, a basis for three main programs: (i) Fpocket, to perform pocket identification, (ii) Tpocket, to organise pocket detection benchmarking on a set of known protein-ligand complexes, and (iii) Dpocket, to collect pocket descriptor values on a set of proteins. Fpocket is written in the C programming language, which makes it a platform well suited for the scientific community willing to develop new scoring functions and extract various pocket descriptors on a large scale level. Fpocket 1.0, relying on a simple scoring function, is able to detect 94% and 92% of the pockets within the best three ranked pockets from the holo and apo proteins respectively, outperforming the standards of the field, while being faster.

**Conclusion:**

Fpocket provides a rapid, open source and stable basis for further developments related to protein pocket detection, efficient pocket descriptor extraction, or drugablity prediction purposes. Fpocket is freely available under the GNU GPL license at .

## Background

In the recent years, in silico structure based ligand design (SBLD) has become a major approach for the exploration of protein function and drug discovery. It has been proven to be efficient in the identification of molecular probes, in investigation of molecular recognition, or in the identification of candidate therapeutic compounds (see for instance [1,2]). Whereas SBLD encompasses a wide range of aspects, one approach of importance is structure based virtual screening (SBVS). In SBVS, one searches, given the structure of a protein, to dock candidate compounds to identify those likely to bind into a candidate ligand binding site (see for instance [3] and references included).

The identification and characterization of pockets and cavities of a protein structure is a key issue of such process that has been the subject of an increasing number of studies in the last decade. Several difficult aspects have to be considered among which: (i) the candidate pocket identification itself [4-26]. Here, one needs methods to identify and delimit cavities at the protein surface that are likely to bind small compounds. (ii) pocket ranking according to their likeliness to accept a small drug-like compound as ligand, for instance. Since often several pockets are detected at a protein surface, it is necessary to have some characterization of them in order to select the relevant ones. Although the largest pocket tends to frequently correspond to the observed ligand binding site (e.g. [18]), this rule cannot be generalised. Different studies have tackled this problem, see for instance [18,19,21,27,28]. It has in particular been shown that the use of evolutionnary information such as residue conservation helps re-ranking the pockets [19,21]. (iii) Last, but not least, there is often an adaptation – the so called induced fit – of the pocket geometry to the formation of a complex with the ligand (see for instance [29-32]). This last point creates several issues in terms of pocket detection – the pocket could or could not be properly detected in absence of ligand – and in terms of scoring since scoring functions are strongly dependent on the quality of the pocket identification and delimitation, but also are sensitive to conformational changes. Here, we focus on the primary but central aspect of candidate pocket identification from structure.

It is not easy to summarise the diversity of approaches that have been proposed so far for candidate pocket identification. Roughly, some are based on pure geometric analysis of the surface of the protein [4-15,18,20,22-26], whereas some others involve energy calculations [16,17]. Another way of distinguishing between the various approaches is to consider the detection algorithms. These can be classified as grid-based, and grid-free approaches. Grid based approaches [4,11,15-17,19,20,23] cover the proteins with a 3D grid and then search for grid points that are not situated within the protein and that satisfy some condition. For instance, POCKET [4], and the derived LigSite [11] search for protein-solvent-protein (PSP) events on the grids to identify pockets as positions enclosed on both sides by the protein. Pocket-Picker [23] uses a buriedness index to identify clusters of grid points likely to correspond to ligand binding pockets. Laurie and Jackson [17] position a methyl probe at grid points and calculate an interaction energy with the protein. An et al. [16] calculate a grid potential map of the Van der Waals force field using a carbon atom probe. Grid free approaches encompass (non exhaustive) probe (or sphere) based approaches as well as methods using the concepts of Voronoi diagrams. Sphere or probe approaches are based on the positioning of probe spheres at protein surface and to identify clusters of spheres having some property representative of candidate pockets. SURFNET [8,21] positions gap spheres between any pairs of atoms, reduces their radii so that they do not intersect any atom, and retains spheres with a radius more than a given threshold. PASS coats the protein using small probes positioned from unique triplets of atoms, and then identifies candidate pockets using a "burial count" – a number of protein atoms within a distance of the probe – to exclude convex parts of the surface. Iterative coating of remaining buried parts further allows the detection of "active site points" that represent the centres of potential pockets. More recently, both Nayal et al. and Kawabata & Go have proposed approaches using two different probe sizes to identify cavities. Small probes are used to identify a collection of positions at protein surface whereas large probes are used as a means to select the small probes located in depressions at protein surface. Among approaches related to Voronoi diagrams, CAST [13] and APROPOS [10], extract from the Delaunay triangulation of the convex hull the so called alpha-shape – a subset of the triangulation from which Voronoi vertices and edges outside the molecule are omitted. The commercial package SiteFinder [33] uses the concept of alpha spheres – spheres that contact four atoms and do not contain any atom (see concepts) – to identify cavities. Finally, Kim et al. [26] have recently proposed another approach based on the identification of "pocket primitives" from Voronoi diagrams.

In terms of availability, several of these approaches can be accessed via web servers (e.g. [34-36]), but very few packages are available for distribution. Some have been released as binaries (e.g. [14]), and for instance only the recently released PocketPicker [23] and LigSite(csc) [19] are available as open source softwares. There are a lot of research topics for which the availability of a free method can be of interest. Concerning this precise field, a part which is of major interest is development of scoring functions. These functions enable ranking of cavities when compared to each other. They are trained usually on descriptors of the binding pocket. Next, one has to assess rapidly the performance of these scoring functions. Still today, extraction of relevant pocket descriptors as well as assessment of scoring functions is an issue. One generally has to develop automatisation protocols for assessment. Available free tools performing these tasks might fasten discovery in computational binding site and drugability prediction. Besides, there are several scopes in which flexible software adaptation from source code might be required. For instance, the search for catalytic site pockets might differ from the search for protein-protein interaction effectors or carbohydrate-protein binding sites. Finally, speed remains an issue, in a context where the pocketome size keeps increasing. In a general manner, the user should also be able to freely complexify the algorithm, in order to improve its performance and repropose the modifications freely to the scientific community. Thus, fast, accurate and high performing development based on a community willing to share their improvements might lead to a leading edge software package for pocket identification. PocketPicker makes one step in this direction. However, it was developed in Python and is specially adapted for visual purposes within PyMol. Thus PocketPicker seems adapted for punctual visual pocket detection, but not really adapted for large scale evaluations, especially due to execution speed limitations.

In this paper, we introduce a free pocket detection software called fpocket. It is based on the alpha sphere theory, an approach that relies on Voronoi tessellation that is among others the basis of the commercial software SiteFinder available within MOE from Chemical Computing Group [33]. It has several inherent advantages such as computational efficiency, the direct identification of the atoms of the proteins involved in a pocket, and promising possibilities to combine pocket detection and docking using a unified framework [37]. Using this approach, we propose a modular package to organise large scale pocket detection, descriptor extraction and benchmarking.

## Implementation

### Concepts

Fpocket relies on the concept of alpha spheres, introduced by Liang and Edelsbrunner [13] and also used by Chemical Computing Group in the SiteFinder software [33].

Briefly, an alpha sphere is a sphere that contacts four atoms on its boundary and contains no internal atom. By definition the four atoms are at an equal distance (sphere radius) to the alpha sphere centre. Alpha sphere radii reflect the local curvature defined by the four atoms: 4 atoms in a plane would correspond to an alpha sphere of infinite radius, and conversely, 4 atoms packed at the apex of a tetrahedron would lead to a value of radius close to that of the Van der Waals radius. For a protein, very small spheres are located within the protein, large spheres at the exterior, and clefts and cavities correspond to spheres of intermediate radii. Thus, it is possible to filter the ensemble of alpha spheres defined from the atoms of a protein according to some minimal and maximal radii values in order to address pocket detection. In practice, alpha sphere identification can be related to Voronoi decomposition of space: the centre of alpha spheres correspond to Voronoi vertices – points at which Voronoi regions intersect.

Once having identified a filtered ensemble of alpha spheres, another property of interest is that candidate regions of interest such as clefts at protein surface have larger occurrence of alpha spheres. Thus, the search for ligand pockets can be turned as the search for clusters of alpha spheres of proper radius. Finally, the knowledge of the spheres also comes with the identification of the atoms of the protein involved. It is thus easy to type the spheres according to some properties depending on the atomic types – such as for instance hydrophobicity – in order to filter the clusters. Conversely, knowing a pocket, it is also possible to extract properties for the atoms defining it.

### Algorithm

The fpocket core can be resumed by three major steps. During the first step the whole ensemble of alpha spheres is determined from the protein structure. Fpocket returns a pre-filtered collection of spheres. The second step consists in identifying clusters of spheres close together, to identify pockets, and to remove clusters of poor interest. The final step calculates properties from the atoms of the pocket, in order to score each pocket.

#### Voronoi tessellation and alpha sphere detection

Voronoi tessellation is performed using the qhull package and more precisely the program qvoronoi [38]. Qhull's source code is freely available on . Fpocket submits the heavy atom set for Voronoi tessellation to Qhull. In return Fpocket receives a set of coordinates of Voronoi vertices, atomic neighbours and vertex neighbours. This list of Voronoi vertices is then pruned according to two parameters: a maximum size of alpha spheres and a minimum size. Pruning the alpha spheres set by this maximum size and minimum size enables the elimination of solvent inaccessible alpha spheres and too exposed alpha spheres. Finally, only alpha spheres defined by zones of tight atom packing are retained and all the other alpha spheres are discarded.

Alpha spheres are then labelled according to the atom type they contact. Fpocket defines alpha spheres as apolar when they are contacting at least 3 atoms with a low electronegativity (< 2.8), typically carbons and sulfur in proteins. Subsequently, polar alpha spheres contact 2 or more polar atoms (typically oxygen or nitrogen).

#### Clustering of alpha spheres

This step has to be performed on several tenth of thousands of alpha spheres. Three different clustering steps are applied to the set of alpha spheres. The first one is a rough segmentation pass. In order to perform this step in a reasonable calculation time, fpocket uses the neighbour lists output from Qhull that indicates Voronoi vertices connected to each other by an edge. Fpocket checks if these interconnected vertices are close to each other and identifies a first set of clusters using a simple distance criterion. After this first pass, all clusters having only one sphere – generally large spheres situated at the protein surface – are removed, and the centre of mass of each cluster is calculated. The next clustering step consists in the aggregation of clusters having proximate centres of mass. This way, small clusters of alpha spheres, especially on the surface are aggregated into one single cluster. Reducing complexity of an alpha sphere cluster on one single barycentre provides a rapid approach in order to group small clusters together, without performing a loop on all alpha spheres. Finally, a step based on a multiple linkage clustering approach is carried out in order to perform final fine clustering. During this step, all vertices of one cluster are compared to vertices of another cluster. If a certain number of alpha spheres of one cluster are near a certain number of alpha spheres of another cluster, both clusters are merged together.

After these three clustering steps, a pruning of uninteresting alpha sphere clusters can be performed. At this stage, small and essentially polar clusters can be dropped from the protein surface. User defined minimum number of alpha spheres and apolar spheres are used in order to influence removal of rather hydrophilic or small putative binding pockets. Note that this facility proposed to users is not used in the present study.

#### Characterization and ranking of the pocket

Last, clustered pockets were characterised in order to rank pockets according to their ability to bind small molecules. Note that the current ranking of pockets does not reflect drugability. It simply reflects the putative capacity of the pocket to bind a small molecule, that might be drug-like, but might also be a sugar, cofactor or coactivator. This rather basic but successful scoring scheme was derived using Partial Least Squares (PLS) fitting to some of the currently implemented pocket descriptors in fpocket.

### Core programs

**Figure 1:**
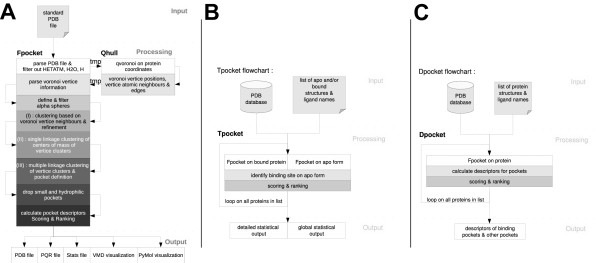
**Fpocket (A), Tpocket (B) and Dpocket (C) flowcharts**.

The fpocket package is made of three components: fpocket (Finding pockets) to perform the pocket identification, as described previously. Tpocket (Testing pockets) is provided in order to organise the benchmarking of the pocket detection algorithm over collections of structures, and dpocket (Describing pockets), designed to extract descriptors from collection of pockets from multiple structures. A flowchart of each is reported figure 1. Note, that the core of tpocket and dpocket is fpocket, exactly the same as the standalone fpocket program. Simply a layer of large scale statistical analysis was added to these two programs, in order to facilitate high throughput pocket detection and assessment of scoring performance.

#### Fpocket

Figure 1a illustrates the workflow of Fpocket (finding pockets), as well as the structure of the input and the output. This program will take as input a protein structure (PDB format) or a list of pdb files and return information about candidate pockets, numbered by rank. Fpocket will usually discard all atoms of the input file tagged as hetero atoms (including solvent and ligands). Nevertheless, cofactors like hemes should be kept during cavity detection, as they are usually part of the functional unit of a protein. Thus, fpocket maintains a list of cofactors accepted as part of the protein during pocket detection. The algorithm is controlled by several parameters that can be adjusted by the user:

- Alpha sphere filtering parameters are related to the minimum (resp. maximum) size of alpha spheres: the minimum (resp. maximum) size an alpha sphere might have during alpha sphere docking on Voronoi vertices. Alpha spheres beneath (resp. above) this size are discarded from clustering.

- Alpha sphere clustering parameters: Three parameters control the three consecutive clustering steps of fpocket: (i) the maximum distance between Voronoi vertices for the 1st clustering step, (ii) the maximum distance between two cluster centroids for clustering step 2, and (iii) the maximum distance between two alpha sphere centres (Voronoi vertices) for the multiple linkage clustering step.

- Pocket pruning parameters: It is controlled by three parameters: (i) The minimum number of alpha spheres in a putative binding pocket, to prune too small clusters, (ii) the minimum ratio of apolar alpha spheres over the total number of spheres to prune too hydrophilic pockets – currently not in use.

On exit, fpocket will return different files containing information about the identified pockets. First, it will return a PDB file containing all atoms used for pocket detection from the input PDB file (ligands are discarded on input unless explicitely notified), supplemented by the positions of all alpha sphere centres (Voronoi vertices) retained after pocket detection. Voronoi vertice positions are added as HETATM in the PDB file. The residue name associated to these vertices is STP (for SiTePoint). Residue numbers are given according to the pocket numbering and thus ranking. One can distinguish two types of Voronoi vertices (encoded by the atom type column of the PDB convention) in the PDB output: (i) APOL, for apolar vertices and (ii) POL, for polar vertices. Second it returns a file using the PQR convention that contains only the alpha sphere centres and radii. Again, residue numbers correspond to pocket ranks. Third, a file containing statistics about each pocket is provided. It lists different characteristics and scores of pockets identified on the surface of the protein. Fourth, scripts are provided, intending to render easier visualisation of putative binding pockets using PyMol or VMD. Finally, a per pocket series of files is also provided. For each pocket, a PDB file containing only the atoms defining the pocket and a PQR containing only the alpha spheres of the pocket are written.

#### Tpocket

Tpocket (Testing pockets) has been designed as a framework for the evaluation of the performance of the pocket detection algorithm and the accuracy of the implemented scoring function: Users trying to implement their own scoring functions can easily assess their performance using tpocket. The general workflow of this framework is presented on figure 1b. Generally one wants to assess a scoring function on a collection of PDB structures for which the binding site is known. In addition, it can be of importance to compare the performance of pocket detection for both apo and holo forms of the same protein. Tpocket can manage both constraints using an input list file, where each line should contain the information about one pair of related apo/holo structures: "path_to_the_apo_structure path_to_the_bound_structure name_of_the_ligand " and the name of the ligand is specified using the same 3 letter code (residue name) as in the PDB file. Note that when assessing the performance of fpocket using a set of apo/holo structures, the two forms should be superposed prior to the analysis.

The tpocket output is split up in two files. First, global performance for all available evaluation criteria described later is provided in a simple text file. Second, more detailed information about pocket detection is written in a separate text file for each structure, including the total number of pockets retained, all evaluation criteria implemented, the rank of the actual pocket detected by fpocket for each criteria, and some other values such as ligand and pocket volume evaluation, number of atoms in the pocket... Among other things, this file allows the identification of structures for which the fpocket detection failed (either because the pocket found has a low rank or was not found at all) for each evaluation criteria.

The fpocket prediction performance presented in this paper are based on tpocket results. Consequently, besides careful manual inspection of pocket evaluation results, they were further validated by an external evaluation script. A SVL (Scientific Vector Language) script was developed using the Moe Software from the CCG. This script evaluates fpocket performances based on fpocket output only. Tpocket and the SVL script gave both exactly the same result.

#### Dpocket

Dpocket (Describing pockets) is designed to organise descriptor collecting from a series of co-crystallized complexes. It accepts a list of structures to analyse using a file containing the information about one structure per line, on the form:

"path_to_the_structure name_of_the_ligand1"

For each structure, dpocket extracts several simple descriptors using atom, amino acid and alpha sphere information. Currently, the set of descriptors implemented is related to (i) alpha spheres (number, polarity, density, ...) (ii) protein atoms (electronegativity, ...) (iii) residues (residue type occurrences, hydrophobicity, ...) (iv) volume. Additionally, some of these descriptors are normalised between 0 and 1 to allow comparison between pockets of different proteins. Although many of these descriptors are basic, users can easily implement more sophisticated analysis of pocket properties. Besides, the current scoring function shows impressive performance and is based on only 5 of these simple descriptors.

Dpocket provides three different output files. First, descriptors extracted from alpha spheres next to the ligand, are written in a separate text file. Second, descriptors for correctly identified binding pockets are extracted. Last, descriptors for other pockets found by fpocket are extracted in a separate text file. Detailed information on each descriptor used in the current version can be found in the full documentation.

### Parameter optimisation

In order to determine optimal parameters for fpocket, a data set based on the protein test set used by An et al. in 2005 [16] for the evaluation of PocketFinder was used. The set described by An et al., composed of 5616 protein ligand complexes and 11510 apo forms is rather redundant, despite the fact that 5616 complexes are composed of the combination of 4711 unique proteins and 2175 unique ligands. The structural redundancy was eliminated allowing a maximum sequence identity of 50% between different proteins of this set. The PDB blastclust file, available on the PDB website was used for this purpose . This first filter resulted in 307 proteins that we further validated by hand, in order to perform training on well defined binding pockets. Monomers and homo-multimere containing more than one single binding pocket for the same ligand were removed. No particular filters were applied to the ligand type, as the druglike concept is still a matter of debates. During training, all hetero atoms were dropped from the PDB structure and pocket detection was performed not taking into account hydrogen atoms. Only structurally important HETATM recordings, like hemes, zinc etc. were kept in order to detect a "biologically" available binding pocket. A complete list of kept HETATM recordings is available in fpocket manual.

Currently, fpocket contains standard parameters determined by an semi combinatorial/empirical optimisation step using this training set. Basically, the fpocket parameters allow enough flexibility to obtain many small pockets as well as few very large pockets. During this optimisation, our goal was to clearly identify the pockets using two main pocket identification criteria (e.g. a good ligand coverage and a low distance value according to the PocketPicker distance criterion). Pockets found by the algorithm should be neither too small nor too large. To do so, it was intended to obtain a good relative overlap (e.g. size of the pocket found by fpocket/size of the actual pocket). Additionally, we attempted to minimise the number of pockets returned by the algorithm. The resulting fpocket standard parameter values are an alpha sphere minimum (resp. maximal) size of 3.0 (resp. 6.0) Å, a minimum connection distance 1 (resp. 2, 3) of 1.73 (resp. 2.5 and 4.0) Å, a minimum number of alpha spheres of 35.

### Scoring function

Fpocket currently uses a simple 3 component PLS derived scoring function. This scoring function makes use of the ligand coverage as the dependant variable, and of the five following descriptors implemented in dpocket as independent variables: (i) the normalised number of alpha spheres, (ii) the normalised mean local hydrophobic density, (iii) the normalised proportion of apolar alpha sphere, (iv) the polarity score (sum of polarity over all amino acids involved in a given pocket using a binary scheme, e.g. 1 for polar, 0 for non polar) and (v) the alpha sphere density, defined as the mean value of all alpha sphere pair to pair distances in the binding pocket.

Note that the normalisation here means that the basic descriptor was scaled to a 0–1 range, so that for example the largest and the smallest pocket within a given protein would have a normalised number of alpha spheres of 1 and 0, respectively.

The model was trained using the dpocket output statistics run on the training dataset previously defined. No additional normalisation of descriptors (such as mean centring) was used as no difference was shown in terms of prediction accuracy.

### Site identification assessment

In order to assess pocket prediction performance, one has to compare identified pockets to the real binding pocket. Different approaches exist in order to do so. Fpocket implements different methods to assess whether a binding pocket was found or not.

PocketPicker criterion (PPc): This is the criterion used in the PocketPicker [23] study. Here the geometric centre of the pocket is calculated. If the position of this centre is within 4 Å from any atom of the ligand, the binding site is considered correctly identified.

**Figure 2:**
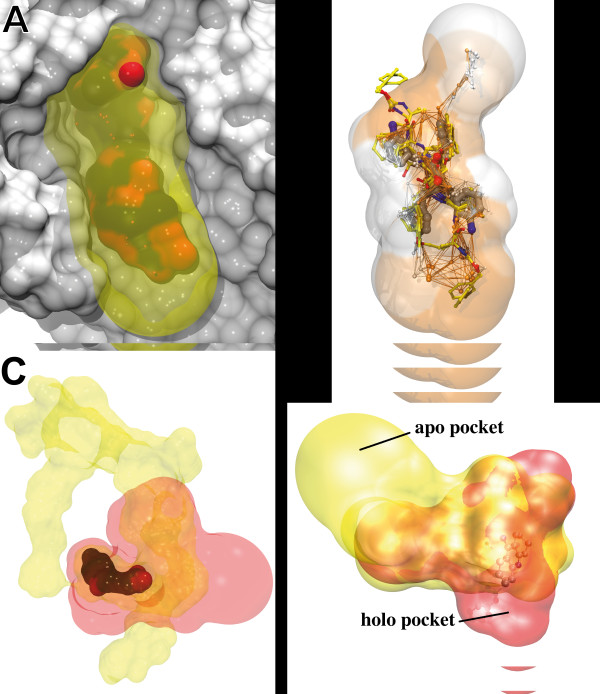
**Examples of pocket detection using fpocket**. **top left**: Rank 1 pocket on the alpha amylase (7TAA). Acarbose in surface/coloured/opaque representation, the binding site is represented as yellow transparent hull. Alpha sphere centres are depicted as small red points. **top right**: Rank 1 pocket of the HIV1 Protease DMP450 complex (PDB Code: 1DMP). DMP450 is depicted in grey CPK representation and the binding pocket as transparent hull. Superposed are other known inhibitors (yellow) binding in the same pocket (PDB Codes: 1Z1H, 2UY0, 2P3B). Alpha sphere centres are depicted as small interconnected spheres. Alpha spheres and the pocket are coloured according to polar (orange) and apolar (white) character. **bottom left**: Cyclooxygenase-2 indomethacin binding site: (red) pocket identified by fpocket,(yellow) pocket identified by PocketPicker. **bottom right**: Acetylcholinesterase rank 1 predicted binding pocket by fpocket. Red: pocket of the holo structure with tacrine (1ACJ), yellow: pocket of apo structure (1QIF). Pockets are represented as a hull resulting from the union of the alpha spheres.

Mutual Overlap criterion (MOc): This criterion considers a pocket successfully identified if at least 50% of the ligand atoms lies within 3 Å of at least one alpha sphere, AND if at least 20% of the pocket alpha spheres lie within 3 Å of the ligand. In other words, the first condition ensures that the ligand is at least half covered by the pocket, and the second condition allows the pocket to be quite large, but not too much as a significant proportion of probe still has to lie next to the ligand. Note that pockets larger than the effective region of interaction with the ligand have to be considered since several ligands may bind to different regions of the pocket (see Figure 2).

The MOc is introduced for two main reasons: (i) to further validate fpocket and see if it's performance remains acceptable using a rather different evaluation criterion and (ii) to address two issues related to the PPc.

Firstly, PPc does not ensure that a reasonable fraction (e.g. one half) of the ligand lies within the pocket identified. For example, a small cluster of probes (alpha spheres for fpocket) next to the ligand could be considered as a successful identification of the pocket even if none of the ligand atoms actually lies within the pocket volume. Secondly, large pockets generally cannot be considered as successfully identified using this criterion. Although it ensures that very large pocket (e.g. the whole protein), are considered as failure, we believe that this criterion is too restrictive, especially (i) when the ligand is small and/or not located at the centre of the pocket found, (ii) when the pocket is simply very large (large protein, multimer...) and (iii) when the pocket does not have a simple globular form.

**Figure 3:**
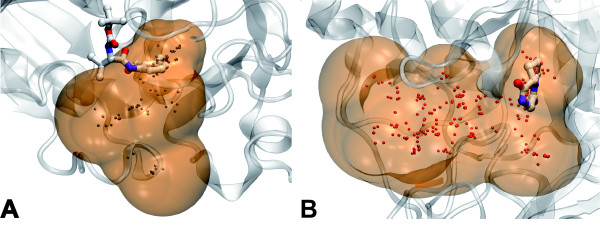
**Pocket detection limits**. **Left**: Example of PDB entry 1esa. A large part of the ligand is outside the pocket detected by fpocket. Despite this fact, a criterion such as the PocketPicker criterion would accept the pocket as successfully identified, and the Mutual Overlap criterion not. **Right**: Example of PDB entry 1w1p. The identified pocket is large compared to the ligand. Its centre of mass is too far from any atom of the ligand for the Pocket Picker criterion to accept it as successfully identified. Ligands are represented using a ball and sticks representation. Alpha sphere centres are represented as small spheres, and their envelope is depicted in brown.

Figure 3 illustrates differences between the two criteria. Here, pockets are considered as successfully (1esa PDB entry) and unsuccessfully (1w1p PDB entry) identified by PPc, respectively. However, for the 1esa case, one cannot consider the pocket as successfully identified, as only a small part of the ligand is covered by the pocket; the MOc considers this case as a failure since less than 50% of the ligand is covered by the pocket. For 1w1p, PPc fails, mainly because the ligand is not located at the centre of the pocket, and because the pocket is rather large; the MOc considers this case as a successful one, as the ligand is covered at 100% and 25% of the alpha spheres lie next to the ligand.

## Results

### Evaluation of pocket prediction accuracy

Table 1 presents fpocket performance on 3 different data sets. The first one consists in a collection of 48 proteins [23] already used in a previous study for which results of several methods on the bound and unbound conformations are reported. In order to keep the comparison valid, we haven't modified this dataset, although we identified several cases of multiple binding sites that should be removed in a rank-based evaluation. The second one was derived from a contribution by Alan C Cheng & al. [39]. They used a set of 63 structure representing 27 pharmaceutical targets, including 23 targets with marketed drugs or drugs in Phase II or above. We have selected randomly one protein-ligand complex for each of these targets to avoid redundancy, and the same filters as those used or the parameter optimization set were applied, resulting in a set of 20 pdb files. Finally, the recently defined Astex diverse set [40] was used. This dataset consists of 85 diverse high resolution protein-ligand crystal structures retrieved from the PDB using newly developed analysis and classification techniques. This last dataset has been built using the following filters: (i) the ligand is drug-like; 23 of the ligands are approved drugs and 6 are currently in clinical trials (ii) no particular target is represented more than once (iii) the proteins are all drug discovery or agrochemical targets (iv) only high quality structures are included for which the ligand electron density supports the entire ligand binding mode (v) no structures are included where the ligand is in contact with protein atoms of crystal symmetric units After applying our filtering procedure, 82 proteins were kept. For sake of comparison, results obtained using the Pocket Picker criterion (PPc) are first discussed. From the proteins in complex with the ligand, fpocket correctly identifies 83% (resp. 92%) of the actual pockets within the top 1 and top 3 ranked pockets, a performance better than other approaches. From the unbound conformations of these proteins, the corresponding results are of 69% and 94%, respectively. At rank 1, similarly to other approaches, fpocket performance decreases, but remains however better than all methods evaluated on this dataset, except LIGSITE (csc) that shows a slightly better performance (2%) and PocketPicker for which fpocket reaches similar score. At rank 3, fpocket outperforms by far all other approaches except possibly LIGSITE (csc) for which no result at this rank is available. This would indicate that fpocket's pocket detection is particularly efficient, and that further filtering on pocket drugability (for instance) could be used to re-rank the top 3 pockets. In order to test the robustness of fpocket depending on the dataset, we also present the results of fpocket and Pocket Picker on two other sets. At rank 1, we observe for fpocket scores of 75 and 67% on the Cheng and Astex diverse sets, respectively. Fpocket scores better than Pocket Picker by 5 and 8% respectively. In addition, again one could note that the fpocket performance at rank 3 remains by far higher.

**Table 1:** Fpocket performance

Dataset	Algorithm	Rank 1		Rank 3	
		unbound	bound	unbound	bound

Pocket Picker					
	Fpocket	69 (67)	83 (85)	94 (92)	92 (92)
	PocketPicker	69	72	85	85
	LIGSITE(CS)	60	69	77	87
	LIGSITE	58	69	75	87
	CAST	58	67	75	83
	PASS	60	63	71	81
	SURFNET	52	54	75	78
	LIGSITE(CSC)	71	79	-	-

Cheng et al.					
	Fpocket	-	75 (70)	-	95 (90)
	PocketPicker	-	70	-	80

Astex Diverse set					
	Fpocket	-	67 (73)	-	82 (88)
	PocketPicker	-	59	-	67

Comparison of results obtained for fpocket and other approaches. For sake of comparison, scores are reported using the PPc, and we present scores at rank 1 and 3 (true pocket in the top 3 pockets proposed by fpocket). For the Pocket Picker dataset, results are taken from [23] for all but fpocket. For fpocket, numbers within parentheses correspond to scores obtained using the MOc.

In Table 1 are also listed the fpocket performances using the mutual overlap criterion (MOc) introduced in this paper. Compared to the PPc, no significant differences are observed in terms of performance measures for the Pocket Picker set, but slightly smaller (resp. better) performance measures on the Cheng (resp. Astex diverse) set. However, on average, the performance at rank 3 remains more stable, close to 90% using the MOc. Looking more in detail, the 5% difference of observed for the Cheng set only represent one protein, due to the low number of structures in this set. The Astex diverse set contains 6 proteins for which the MOc and PPc disagree, and for all of them, the MOc detects the pocket correctly while the PPc does not. Pocket size seems to be the major issue. In the Astex dataset, the mean number of atoms per pocket is 91 (defined as all unique atom contacted by alpha spheres within the pocket). For the 6 cases mentioned previously, this number ranges between 116 and 281. This illustrates a better behaviour of the MOc on larger pockets for which PPc seems unadapted – see methods.

### Examples of successful identification of binding sites

Figure 2a shows the successful identification (rank 1) of the acarbose binding pocket on alpha amylase (PDB code 7taa). Acarbose is represented in coloured surface and the fpocket identified binding pocket as transparent hull around the ligand. This rather long and large pocket has a buried and a more solvent exposed part. Despite this heterogeneity within the whole binding pocket, fpocket identifies the whole pocket with a reasonable pocket volume around the ligand.

On figure 2b another interesting feature about fpocket is shown. Here the binding pocket of HIV1 protease is depicted in complex with the Dupont Merck inhibitor DMP450 (PDB code 1dmp). For representative reasons the protein structure was omitted and only the surface of the pocket is shown (alpha sphere surface) with the embedded ligand. The small interconnected spheres are the alpha sphere centres. Orange alpha spheres are polar alpha spheres, white alpha spheres are apolar. The same colour code was used for the colouring of the pocket surface. Here, one can notice that the positions of alpha sphere centres follow surprisingly well the topology of the ligand (grey). Note, however, that this is not a general property of Voronoi vertices. Next, physicochemical properties of the ligand are reflected by the sourrounding binding pocket. The pocket identified by fpocket seems far longer than the actual binding position of the ligand. However other drug like molecules (yellow) are known to make interactions also with residues situated on the edge of the pocket (top and bottom here).

These examples show that fpocket is able to detect solvent exposed and very buried binding sites, that bind ligands of a very different nature (oligosaccharide, drug)

Last an example of cyclooxygenase-2 indomethacin complex (PDB code 4cox) is depicted on figure 2c. The binding pocket identified using PocketPicker is represented as yellow halo. As red halo one can find the fpocket identified binding pocket. Both binding pockets include the actual space of the pocket occupied by the ligand, but the PocketPicker yields a far bigger pocket, including sourrounding channels.

### Examples of unsuccessful identification of binding sites

Figure 2d depicts one example of a binding site that was not correctly identified according to the PPc (see methods). These structures are part of the PocketPicker data set. Here the acetylcholinesterase active site gorge was successfully identified and ranked on the holo form (PDB code: 1acj). The pocket is represented as red envelope. The same pocket on the apo form (PDB code: 1qif) depicted in yellow in figure 3a shows a completely different shape compared to the holo form. This is due to the fact, that the binding pocket is very buried and upon closure of the binding site entry a longer binding pocket was identified. According to the PocketPicker criterion fpocket did not identify well the pocket in the apo form, although the identified binding pocket overlaps nearly completely the previously identified holo pocket. This example shows the limits of the criterion used by PocketPicker to distinguish correctly identified binding sites from others. The MOc overlap criterion presented here and used in similar ways in other studies shows better accordance to visual results than the simple distance criterion used by PocketPicker.

### Computational performance

The algorithm was assessed on a Intel Celeron M 1.6 Ghz, 1 Gb RAM architecture and performed roughly one structure per less than one to three seconds, depending on the size of the structure. For the sake of completeness, performance of LigSite and PASS was compared on the same structures. LigSite performed pocket detection on one structure in 5 seconds, PASS in 4 to 5 seconds. Thus, fpocket appears particularly well suited for large scale evaluations and is situated among the fastest algorithms in the field.

PocketPicker performs roughly one structure in several hours of calculation depending on the size of the structure.

## Conclusion

We have introduced fpocket, a new open source pocket identification platform. Compared to other approaches of the field, Fpocket performs well on state of the art data sets. From the complexed protein conformations, fpocket reaches the best performance at rank 1. On the ligand free structures, similarly to other approaches, fpocket performance drops at rank 1, but is much better at rank 3, outperforming other approaches by more than 9%, opening the door to further pocket drugability filtering approaches. Interestingly, fpocket is among the fastest algorithms in the field. This makes fpocket particularly well suited for high throughput pocket detection and construction of cavity databases. Next, fpocket comes with its underlying programs, tpocket and dpocket, providing powerful research tools for a large scale assessment of own pocket scoring functions and properties of binding pockets, respectively. Its open source character provides a useful contribution to the scientific community willing to further develop and research in the pocket identification and specific molecular binding field.

## Availability and requirements

Fpocket source code (Linux) is freely available under the GNU GPL license at . The required Qhull package is shipped and compiled together with fpocket in the official fpocket release.

## Authors' contributions

VLG and PS have equally contributed to fpocket development. PT has initiated and supervised fpocket development.
